# Retinopathy of Prematurity and Bronchopulmonary Dysplasia are Independent Antecedents of Cortical Maturational Abnormalities in Very Preterm Infants

**DOI:** 10.1038/s41598-019-56298-x

**Published:** 2019-12-23

**Authors:** Julia E. Kline, Venkata Sita Priyanka Illapani, Lili He, Mekibib Altaye, Nehal A. Parikh

**Affiliations:** 1 Perinatal Institute, Cincinnati Children’s Hospital Medical Center, Cincinnati, OH USA; 2 Department of Pediatrics, University of Cincinnati College of Medicine, Cincinnati, OH USA; 3 Center for Perinatal Research, The Research Institute at Nationwide Children’s Hospital, Columbus, OH USA; 4 Divison of Biostatistics, Cincinnati Children’s Hospital Medical Center, Cincinnati, OH USA

**Keywords:** Neonatal brain damage, Retinopathy of prematurity

## Abstract

Very preterm (VPT) infants are at high-risk for neurodevelopmental impairments, however there are few validated biomarkers at term-equivalent age that accurately measure abnormal brain development and predict future impairments. Our objectives were to quantify and contrast cortical features between full-term and VPT infants at term and to associate two key antecedent risk factors, bronchopulmonary dysplasia (BPD) and retinopathy of prematurity (ROP), with cortical maturational changes in VPT infants. We prospectively enrolled a population-based cohort of 110 VPT infants (gestational age ≤31 weeks) and 51 healthy full-term infants (gestational age 38–42 weeks). Structural brain MRI was performed at term. 94 VPT infants and 46 full-term infants with high-quality T2-weighted MRI were analyzed. As compared to full-term infants, VPT infants exhibited significant global cortical maturational abnormalities, including reduced surface area (−5.9%) and gyrification (−6.7%) and increased curvature (5.9%). In multivariable regression controlled for important covariates, BPD was significantly negatively correlated with lobar and global cortical surface area and ROP was significantly negatively correlated with lobar and global sulcal depth in VPT infants. Our cohort of VPT infants exhibited widespread cortical maturation abnormalities by term-equivalent age that were in part anteceded by two of the most potent neonatal diseases, BPD and ROP.

## Introduction

Worldwide, preterm birth affects slightly over 10% of pregnancies^1^. Although the survival rate has increased dramatically, preterm-born infants remain at high risk for neurodevelopmental impairments (NDI). Infants born very preterm, at less than 31 weeks gestational age (GA), often develop NDI such as cognitive, behavioral, and psychological abnormalities^2–4^. Many also develop motor impairments, including the 10% who develop cerebral palsy^5^.

On structural magnetic resonance imaging (MRI) at term-equivalent age (TEA), preterm infants exhibit white matter signal abnormalities, destructive abnormalities, and global and regional brain tissue volume decreases that are associated with NDI^6–8^. Changes in cortical maturational features may also be early biomarkers of NDI. Premature infants have less cortical surface area than full term controls^9^, which is correlated with diminished cognitive ability later in life^10–12^. Preterm infants also have altered cortical folding, which can be assessed via a number of metrics. For instance, gyrification index, or the ratio of cortex within the sulcal folds to cortex on the outer brain surface, is decreased in preterm infants compared to term controls^13,14^. A complementary folding metric, sulcal depth, tends to primarily decrease^13–15^ in preterm groups. Curvature of the inner cortical surface/outer white matter is also reportedly increased in preterm infants^16^. Given that a significant amount of cortical expansion and folding occurs in the third trimester^17^, these alterations are likely caused by early exposure to the ex-utero environment^16,18^ and the secondary illnesses and interventions that premature babies are at risk for^13,19^.

One such secondary illness, bronchopulmonary dysplasia (BPD) is a chronic lung disease that affects mostly preterm-born infants who require prolonged mechanical ventilation or continuous positive airway pressure and supplementary oxygen for respiratory distress syndrome^20^. BPD has been linked to adverse neurodevelopmental outcomes such as cerebral palsy^21^, cognitive deficits^22^, language deficits^23,24^, and many combinations thereof^25–27^. Another secondary illness, retinopathy of prematurity (ROP) is a potentially blinding eye disease, which is also found predominantly in premature infants and has been linked to the overuse of oxygen and early illness severity. It has been suggested that ROP is more than just an eye disease and is related to pathology of both the retina and the neurovasculature^28^. Severe ROP is associated with delayed white matter maturation, poorer cognitive and motor scores at 18 months^29^ and lower IQ in adolescence^30^. There is evidence that BPD and ROP tend to co-occur, but they are independently predictive of neurodevelopmental impairment^27^. For instance, in a large population-based study, the combination of BPD, ROP, and brain injury, strongly and independently predicted the risk of disability in very low birth weight infants^31^. However, no prior studies have linked these two common preterm morbidities with abnormal cortical features at TEA.

Our objectives were: (1) quantify and contrast cortical features between full-term (FT) and very preterm (VPT) infants in 50 brain regions in a large population-based cohort and (2) examine the impact of two of the most potent neonatal diseases, ROP and BPD, on these cortical features, while controlling for known covariates. We hypothesized that (1) preterm infants would show widespread significant alterations in cortical features compared to the full-term control group, namely decreased cortical surface area, gyrification, and sulcal depth and increased white matter curvature and (2) BPD and ROP would be correlated with the objectively-quantified cortical features in the preterm group. These analyses should (1) objectively quantify early alterations in cortical features in premature infants, which may serve as early biomarkers of NDI and (2) shed light on specific secondary illnesses which may exacerbate these alterations.

## Methods

### Subjects

We prospectively enrolled 110 VPT infants from four level III NICUs from December 2014 to April 2016. These NICUs – Nationwide Children’s Hospital, Ohio State University Medical Center, Riverside Hospital, and Mount Carmel St. Ann’s Hospital – cared for approximately 80% of VPT births in the Columbus, Ohio region. We prospectively enrolled 51 healthy FT control infants from September 2014 to August 2015 from the well-baby nurseries of these hospitals. The inclusion criteria for full term infants included a GA of 38–42 weeks, appropriate weight for GA, and a normal well-baby nursery course. Full-term exclusions included congenital/chromosomal anomalies of the brain, spine, heart, or lungs, significant maternal conditions (e.g. insulin-dependent diabetes or severe preeclampsia), intrauterine drug or alcohol exposure, and a history of perinatal distress or complications. Preterm inclusion criteria included a GA of ≤31 weeks, while exclusion criteria included congenital/chromosomal anomalies of the brain, spine, or heart (e.g. Dandy-Walker malformation, myelomeningocele, cyanotic heart disease). We excluded any infants hospitalized at 44 weeks postmenstrual age (unless they were at Nationwide Children’s Hospital, the site of imaging). The Nationwide Children’s Hospital Institutional Review Board approved the study (Protocol#: IRB13-00636). Reciprocity agreements with the Institutional Review Boards of Nationwide Children’s Hospital, Ohio State University Medical Center, Riverside Hospital, and Mount Carmel St. Ann’s Hospital allowed for approval at all sites. The study methods were carried out in accordance with the relevant guidelines and regulations. Written informed consent was obtained from a parent or guardian of all infants.

### Imaging methods

MRI data was acquired on a 3T Siemens Skyra scanner at Nationwide Children’s Hospital at a mean (SD) postmenstrual age of 40.2 (0.6) weeks for the VPT cohort and 41.3 (0.8) weeks for the FT cohort. The majority of infants at Nationwide were imaged while inpatients, and all infants from the other NICUs were imaged after discharge. The scans were overseen by a skilled neonatal nurse and a neonatologist, who monitored heart rate and oxygen saturations throughout. We performed imaging without sedation using these procedures: the infant was fed 30 minutes prior to MRI, silicone earplugs were placed (Instaputty, E.A.R. Inc, Boulder, CO), and a blanket and vacuum immobilization device (MedVac, CFI Medical Solutions, Fenton, MI) helped promote natural sleep. We used these acquisition parameters: axial T2-weighted: echo time 147 ms, repetition time 9500 ms, flip angle 150°, resolution 0.93 × 0.93 × 1.0 mm^3^, scan time 4:09 min. Additional sequences such as T1- and susceptibility-weighted images were acquired for qualitative assessment of abnormality.

### MRI processing

**Figure 1:**
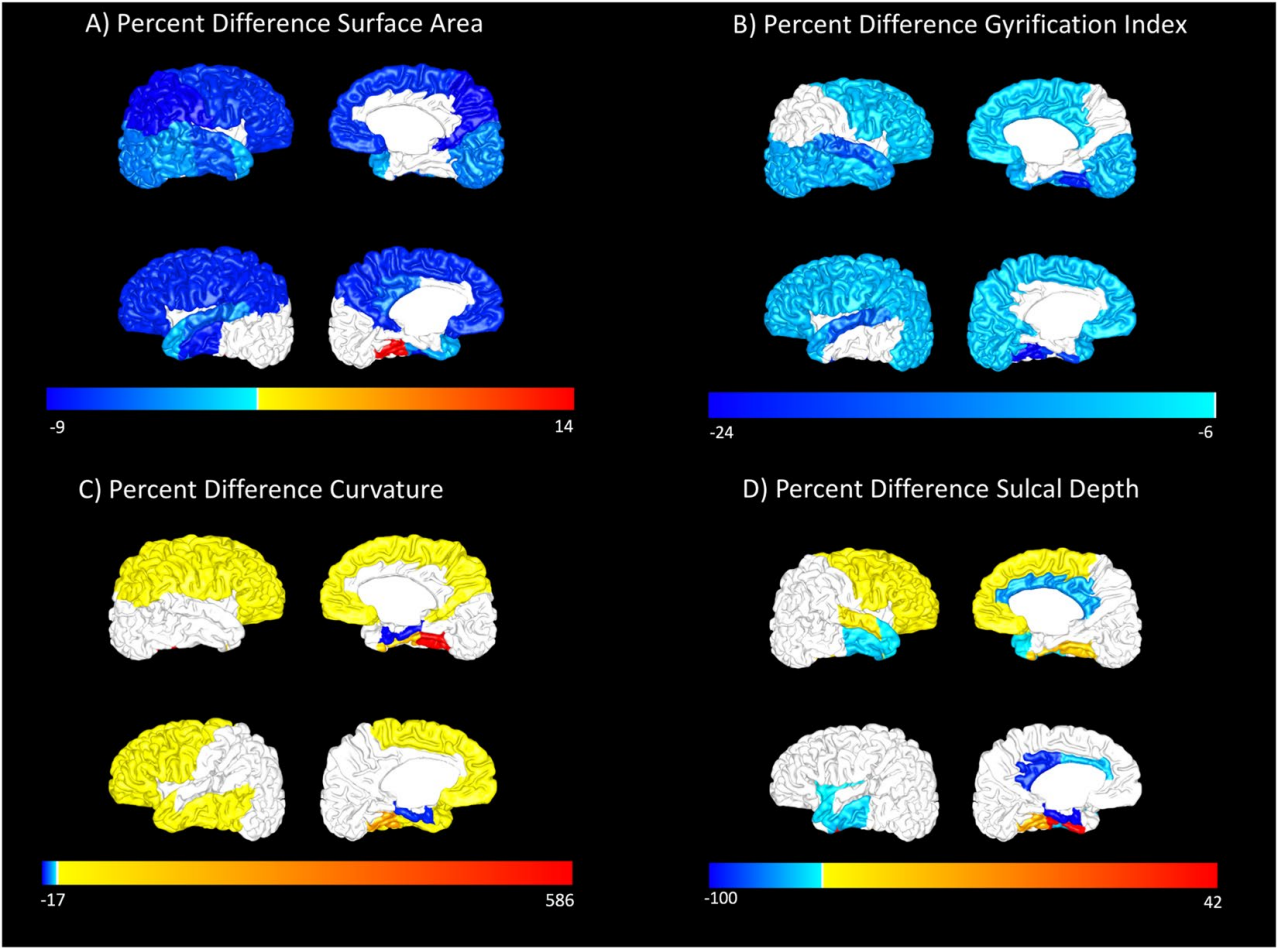
An example T2-weighted MRI scan from one VPT subject is shown in sagittal, coronal, and horizontal views (top row). A segmentation from the Developing Human Connectome Pipeline (dHCP) is overlaid on the original image (bottom row). The dHCP performs cortical and sub-cortical volume segmentation, cortical surface extraction, and cortical surface inflation and was specifically designed for neonatal T2-weighted MRI brain scans.

All T2-weighted images were postprocessed using the developing Human Connectome Pipeline (dHCP)^32^ developed for neonatal MRI, in order to automatically extract global and regional values for cortical features. The dHCP pipeline leverages FreeSurfer for tissue segmentation and volume estimation. It also calculates a value for the cortical features of interest, surface area, gyrification index, sulcal depth, and inner cortical curvature, for each of the 50 regions of the Gousias neonatal atlas^33^ and for the whole brain. All tissue segmentations (Fig. 1) and cortical surface results were visually inspected. Any scan with significant movement artifact, missing cortical boundaries, or moderate to severe ventriculomegaly was excluded at this point, as ventriculomegaly tended to interfere with proper tissue classification by the dHCP.

### Statistical analysis

Our first major statistical analysis assessed significant differences in global and regional cortical features between the full-term and the very preterm groups. We began by transforming non-normal cortical feature values using either log transforms, power transforms, or the Yeo-Johnson transform^34^ (for negative values), as appropriate. We then applied analysis of covariance (ANCOVA) to calculate significant group differences in the global and regional cortical features. We performed the ANCOVA analysis two ways: 1) with postmenstrual age (PMA) at MRI as the only covariate and 2) with PMA at MRI and total tissue volume (TTV) both as covariates. TTV was included to correct for the difference in brain size between the very preterm and the full term infants^35–37^. We did not use total cranial volume as a covariate in any analyses, because the very preterm group had significantly increased ventricular volume, despite the exclusion of subjects with ventriculomegaly. Following the ANCOVA, we used the ‘adjust’ command in Stata to center the covariates on their means and obtain the adjusted group means, which were then transformed back to their original units, if applicable. Because we analyzed so many cortical metrics (>200), we corrected for false discovery rate (FDR) using the Benjamini-Hochberg procedure with a chosen false discovery rate of 5%.

Our second major statistical analysis was a multivariable linear regression analysis examining the impact of BPD and ROP on all four global cortical metrics in the very preterm group, adjusted for covariates. We defined BPD (any severity) per Jobe and Bancalari’s original definition^38^ and ROP using the International Classification of ROP guidelines^39^. Because we hypothesized that BPD and ROP would predict cortical metric values independently of other known clinical or social demographic covariates, we took care to determine the best covariates for each regression. For each regression/global cortical metric, we started with 20 possible covariates (Table 1) from three distinct time periods – the antenatal, intrapartum, and postnatal periods –that had been associated with BPD, ROP, or NDI in previous research. We performed a sequential covariate selection method that has been published on several times previously^37,40,41^. Potential covariates were first tested with only other potential covariates from the same time period. For each individual period, covariates correlated with the cortical metric of interest at p < 0.25 were all entered into backward stepwise regression^42^, and were deemed significant for that period if p < 0.05 in multi-variable regression. Covariates deemed significant from all periods using the above criteria were included in the final model, even if they became non-significant with the addition of other covariates. Overall, this method 1) prevents later-occurring significant covariates from displacing earlier-occurring significant covariates and 2) helps avoid overfitting the model with too many covariates. Known highly-important covariates of brain development, PMA at MRI and injury score on structural MRI^43^, were forced into all final models. Next, we fit all resultant cortical surface metric models for each individual lobe, using multivariate regression in Stata, to determine whether the associations between BPD/ROP and cortical surface metrics were strongly regional.

**Table 1:** Characteristics of the final very preterm and full-term cohorts.

Characteristics	Very Preterm Infants (N = 94)
**Antenatal**	
Chorioamnionitis, n (%)	14 (14.9)
Antenatal steroids, n (%)	82 (87.2)
Male, n (%)	53 (56.4)
Intrauterine growth restriction	7 (7.4)
**Intrapartum**	
Gestational age (weeks), mean (SD)	28.3 (2.5)
Birth weight (g), mean (SD)	1121.7 (394.1)
Head circumference (mm), mean (SD)	25.6 (3.0)
Low NICU admission temperature, n (%)	27 (28.7)
Apgar score at 5 minutes <5, n (%)	18 (19.4)
**Postpartum/neonatal**	
Total parenteral nutrition (days), mean (SD)	18.9 (18.0)
Days of maternal breast milk in first 28 days, mean (SD)	23.5 (6.8)
Late onset culture-positive sepsis, n (%)	13 (13.8)
Patent ductus arteriosus, n (%)	20 (21.3)
Necrotizing enterocolitis, n (%)	10 (10.6)
Caffeine treatment, n (%)	83 (88.3)
Moderate or severe injury on structural MRI, n (%)	9 (9.6)
Surgery for necrotizing enterocolitis and spontaneous intestinal perforation, n (%)	5 (5.3)
Major surgery during NICU stay, n (%)	13 (13.8)
Moderate or severe bronchopulmonary dysplasia, n (%)	48 (51.1)
Retinopathy of prematurity, n (%)	46 (48.9)
Post menstrual age at scan, mean (SD), range	40.2 (0.6), (39.3–43.1)
	**Full-Term Infants (N = 46)**
Male, n (%)	22 (47.8)
Gestational age (weeks), mean (SD)	39.5 (0.8)
Post menstrual age at scan, mean (SD), range	41.3 (0.8), (40.0–43.0)

Finally, we performed a third minor statistical analysis, using Pearson correlation analysis to assess the interrelation between all four global cortical metrics. All analyses were performed in STATA 15.1 (Stata Corp., College Station, TX), except the Benjamimi-Hochberg analysis, which was performed in python.

## Results

Of the 110 eligible VPT infants, one was missing a T2 image, 10 had significant ventriculomegaly that adversely affected brain segmentation. Four T2 images had significant artifact, and one had missing cortical boundaries. The remaining 94 infants were included in the analysis. Of these, only one infant had significant macrostructural injury on MRI. Of the 51 eligible FT subjects, four T2 images had missing boundaries, and one had poor alignment with the dHCP atlas. The remaining 46 were included in the analysis. Table 1 shows the baseline characteristics of our cohorts.

The results of our two ANCOVA group analyses were highly similar, therefore, we will present results controlled for both PMA at MRI and TTV. (Results controlled for PMA only are in the supplement.) Globally, compared to the full-term infants, the very preterm infants showed a 6 to 7% reduction in both surface area and gyrification index and a 6% increase in curvature. Whole-brain sulcal depth was not significantly different between the groups (Table 2).

**Table 2:** Whole-brain mean values and percent differences for four global cortical metrics in very preterm and full-term infants (corrected for PMA at MRI scan and TTV).

Surface Metrics	Very Preterm Infants (N = 94)	Full-term Infants (N = 46)	Relative Percent Difference, Mean (95% CI)	*P*
Gyrification Index (unitless)	2.49 (0.20)	2.67 (0.30)	−6.74% (−9.90%, −3.58%)	<*0.001*
Surface Area (mm^2^)	81210.10 (4527.51)	86252.30 (6836.50)	−5.85% (−8.07%, −3.62%)	<*0.001*
Curvature (1/mm)	2.14 (0.25)	2.02 (0.38)	5.94% (0.68%, 11.20%)	*0.002*
Sulcal Depth (unitless; mean convexity/concavity)	24.54 (3.42)	24.66 (5.16)	−0.49% (−5.39%, 6.36%)	0.84

**Figure 2:**
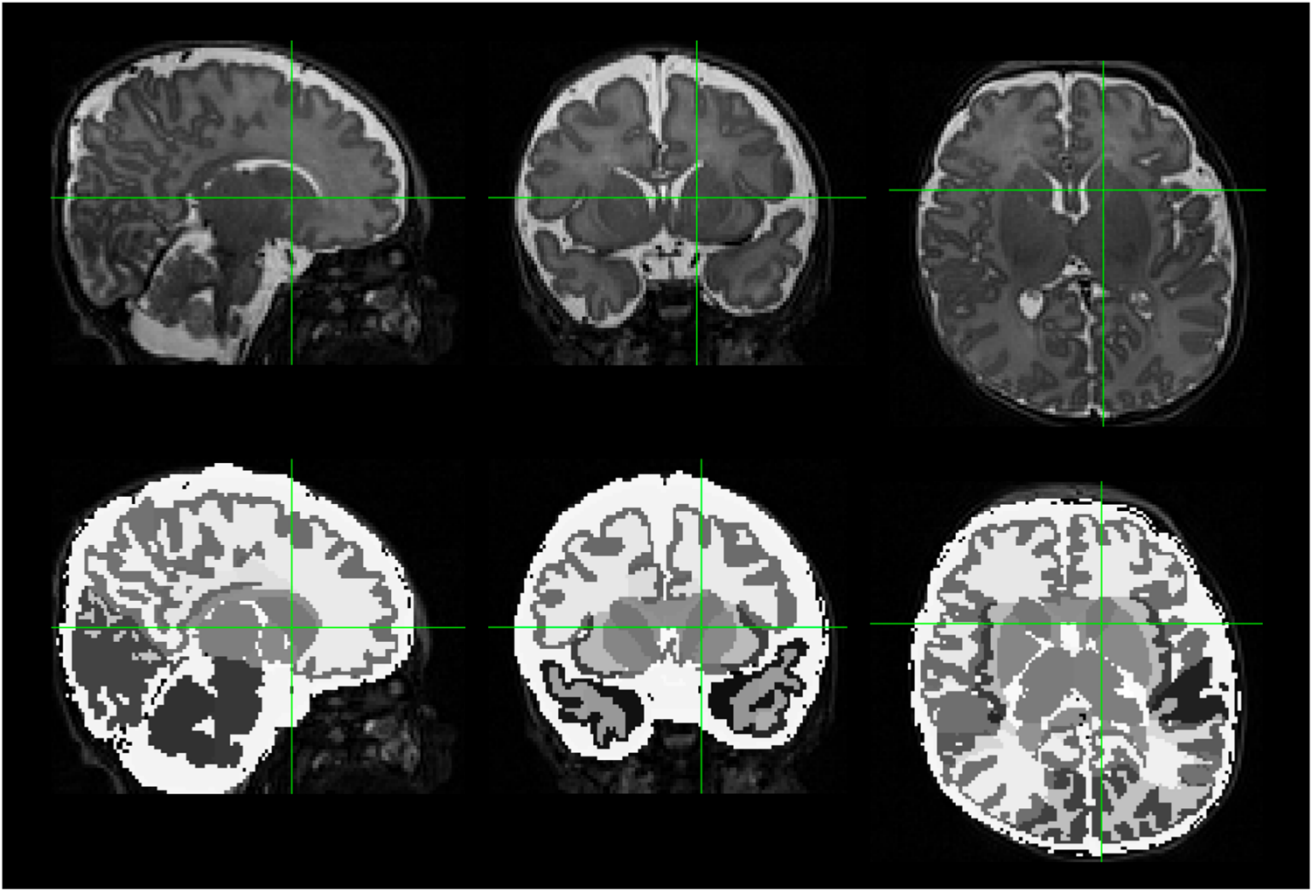
Mean Percent Difference in Cortical Metrics (Full-term to Very Preterm). Regional percent differences in adjusted group means for surface area (panel A), gyrification index (panel B), curvature of the white matter surface (panel C), and sulcal depth (panel D). For regions with significant differences between groups (after false discovery rate correction), percent difference values (VPT − FT)/FT * 100 are projected onto a representative subject brain from the very preterm group. These values have been corrected for postmenstrual age at MRI scan and total brain tissue volume. For each panel, Top row: right hemisphere; Bottom row: left hemisphere.

Regionally, the very preterm cohort had reduced surface area bilaterally in the frontal, parietal, and temporal lobes and in the insula and the right occipital lobe compared to the full-term group. (Range: 3.1% to 9.0%). VPT surface area increased (13.5%) only in the left fusiform gyrus (Fig. 2A). The VPT infants had reduced gyrification in bilateral regions of all lobes (Range: 6.1% to 24.0%.) compared to the full-term group, with the largest decreases in the temporal lobes. (Fig. 2B). Very preterm curvature increased in bilateral regions of the frontal, parietal, and temporal lobes (7.1% to 85.7%.) compared to the full-term group, with extreme values in the bilateral fusiform gyrus (586.0% right; 238.6% left) (Fig. 2C), however given the small size of this structure, its magnitude may be spurious. Curvature decreased in only one region in the VPT group, the bilateral parahippocampal gyrus (16.5% right; 13.8% left). Although there was no significant difference between groups for global sulcal depth, our preterm group had many significant regional sulcal depth differences compared to the full-term group (Fig. 2D) in the frontal, parietal, and temporal lobes. (Range: −100.4% to 41.5%).

Our preterm only regression analysis uncovered correlations between BPD and surface area and ROP and sulcal depth, in bivariable analysis and in multivariable regression adjusted for covariates. For surface area, the significant clinical covariates from the sequential variable selection procedure^37,40,41^ were injury score on MRI, PMA at MRI, sex, intrauterine growth restriction, head circumference at birth, and maternal breastmilk duration within the first 28 postnatal days. In a multivariable model with these covariates, BPD was negatively correlated with surface area (p = 0.009) (Table 3). The significant clinical covariates chosen for sulcal depth were injury score on MRI, PMA at MRI, GA, and birth weight z-score. In a multivariable model with these covariates, ROP was negatively correlated with sulcal depth (p = 0.032) (Table 3). ROP was not significantly correlated with surface area and BPD was not significantly correlated with sulcal depth in multi-variable regression with covariates. Likewise, BPD and ROP were both not significantly correlated with either gyrification index or curvature in multi-variable regression with covariates. Regionally, BPD was significantly negatively correlated with surface area in all lobes of the brain (Table 4). ROP was significantly negatively correlated with sulcal depth in the parietal lobe (Table 5).

**Table 3:** Multivariable Regression Models of Cortical Surface Area and Sulcal Depth.

Clinical Risk Factors	Multivariable regression	*P* value
	Coefficients (95% CI)	
**Surface Area**		
Male Sex	6626.74 (3444.97, 9808.51)	<*0.001*
Intrauterine growth restriction	−8773.05 (−14955.95, −2590.14)	*0.006*
Head circumference at birth	453.60 (−246.23, 1153.43)	0.201
Maternal breast milk duration (days)	327.29 (86.98, 567.61)	*0.008*
Postmenstrual age	3621.56 (1023.68, 6219.43)	*0.007*
Injury score on structural MRI	133.81 (−697.31, 964.94)	0.750
Bronchopulmonary dysplasia	−5508.80 (−9603.97, −1413.64)	*0.009*
**Sulcal Depth**		
Gestational age	0.36 (0.09, 0.62)	*0.009*
Z-score of birth weight	0.49 (−0.19, 1.17)	0.154
Postmenstrual age	0.37 (−0.44, 1.18)	0.364
Injury score on structural MRI	0.13 (−0.14, 0.40)	0.348
Retinopathy of prematurity	−1.44 (−2.76, −0.13)	*0.032*

**Table 4:** Multivariate regression associating BPD with surface area for each individual lobe, after controlling for important covariates.

	Coefficients (95% CI)	P value
Occipital Lobe Surface Area		
Male Sex	384.60 (56.20, 713.00)	*0.022*
Intrauterine growth restriction	−916.76 (−1554.92, −278.60)	*0.005*
Head circumference at birth	10.12 (−62.11, 82.35)	0.781
Maternal breast milk duration (days)	29.09743 (4.29, 53.90)	*0.022*
Postmenstrual age	118.36 (−149.78, 386.49)	0.383
Injury score on structural MRI	5.72 (−80.06, 91.51)	0.895
Bronchopulmonary dysplasia	−548.98 (−971.65, −126.30)	*0.012*
**Parietal Lobe Surface Area**		
Male Sex	658.36 (285.90, 1030.82)	*0.001*
Intrauterine growth restriction	−952.96 (−1676.74, −229.19)	*0.010*
Head circumference at birth	80.90 (−1.03, 162.82)	0.053
Maternal breast milk duration (days)	38.83 (10.69, 66.96)	*0.007*
Postmenstrual age	505.56 (201.45, 809.67)	*0.001*
Injury score on structural MRI	−11.61 (−108.90, 85.68)	0.813
Bronchopulmonary dysplasia	−532.13 (−1011.52, −52.75)	*0.030*
**Temporal Lobe Surface Area**		
Male Sex	660.71 (320.71, 1000.71)	<*0.001*
Intrauterine growth restriction	−791.03 (−1451.72, −130.34)	*0.020*
Head circumference at birth	36.14 (−38.64, 110.92)	0.339
Maternal breast milk duration (days)	35.21 (9.53, 60.89)	*0.008*
Postmenstrual age	403.11 (125.51, 680.71)	*0.005*
Injury score on structural MRI	58.78 (−30.03, 147.59)	0.192
Bronchopulmonary dysplasia	−573.45 (−1011.05, −135.86)	*0.011*
**Frontal Lobe Surface Area**		
Male Sex	1307.21 (740.90, 1873.52)	<*0.001*
Intrauterine growth restriction	−1399.86 (−2500.33, −299.39)	*0.013*
Head circumference at birth	86.16 (−38.40, 210.72)	0.173
Maternal breast milk duration (days)	50.82 (8.04, 93.59)	*0.020*
Postmenstrual age	635.40 (173.01, 1097.78)	*0.008*
Injury score on structural MRI	17.56 (−130.37, 165.49)	0.814
Bronchopulmonary dysplasia	−906.57 (−1635.45, −177.69)	*0.015*

**Table 5:** Multivariate regression associating ROP with sulcal depth for each individual lobe, after controlling for important covariates.

	Coefficients (95% CI)	P value
Occipital Lobe Sulcal Depth		
Gestational age	1.16 (0.15, 2.16)	*0.024*
Z-score of birth weight	2.33 (−0.23, 4.89)	0.074
Post menstrual age	2.30 (−0.75, 5.36)	0.138
Injury score on structural MRI	0.17 (−0.85, 1.19)	0.740
Retinopathy of prematurity	3.36 (−1.61, 8.33)	0.183
**Parietal Lobe Sulcal Depth**		
Gestational age	0.68 (0.09, 1.28)	*0.024*
Z-score of birth weight	−0.19 (−1.70, 1.32)	0.805
Post menstrual age	1.06(−0.74, 2.86)	0.246
Injury score on structural MRI	0.07 (−0.54, 0.67)	0.830
Retinopathy of prematurity	−3.39 (−6.32, −0.46)	*0.024*
**Temporal Lobe Sulcal Depth**		
Gestational age	−0.01 (−0.69, 0.68)	0.981
Z-score of birth weight	0.41 (−1.33, 2.16)	0.639
Post menstrual age	−0.49 (−2.57, 1.59)	0.640
Injury score on structural MRI	0.08 (−0.62, 0.78)	0.824
Retinopathy of prematurity	−0.53 (−3.91, 2.86)	0.758
**Frontal Lobe Sulcal Depth**		
Gestational age	0.41 (−0.15, 0.96)	0.148
Z-score of birth weight	0.18 (−1.23, 1.59)	0.798
Post menstrual age	−0.05 (−1.73, 1.63)	0.953
Injury score on structural MRI	0.12 (−0.44, 0.69)	0.664
Retinopathy of prematurity	−2.48 (−5.22, 0.26)	0.075

Our four global cortical metrics were strongly and significantly interrelated. Sulcal depth had a significant negative relationship with curvature (r = −0.37) and a positive relationship with surface area (r = 0.34). Curvature was significantly negatively correlated with both gyrification index (r = −0.20) and surface area (r = −0.40). Gyrification index was positively correlated with surface area (r = 0.28). Sulcal depth and gyrification index were not significantly correlated.

## Discussion

Our results extend published findings of significant cortical maturation abnormalities in very preterm infants at TEA. Overall, our very preterm group showed decreased global and regional gyrification, decreased global and regional surface area, and increased global and regional curvature compared to the full-term group. Our preterm group also showed regional alterations in sulcal depth compared to the full-term group, with decreases tending to dominate. Unlike prior studies, we also identified two important risk factors – BPD and ROP – for abnormal changes in surface area and sulcal depth, respectively. In prior neuroimaging studies, preterm infants with BPD and ROP have not exhibited consistent structural abnormalities to suggest that ROP and BPD-associated NDI are mediated through qualitative abnormalities visible on structural MRI. Although we cannot determine a definitive causal link, our results suggest that aberrant cortical maturation could be in the causal pathway between severe neonatal illnesses like ROP and BPD and future NDI.

Reduced surface area in preterm subjects is well-documented at TEA^10,13^ and in childhood^11,12^, and reduced gyrification index in preterm subjects is also well-documented at TEA^13,14,44^. Engelhardt *et al*. reported approximately 13% reduced gyrification and 19% reduced surface area in preterm infants compared to full-term infants at TEA, which is larger than our finding (7% reduced gyrification and 6% reduced surface area, globally). However, we excluded subjects with ventriculomegaly or injuries such as intraventricular hemorrhage (27% of the preterm subjects in Engelhardt’s study had intraventricular hemorrhage), and we studied twice as many infants. Zhang reported 4% reduced gyrification index and 10% reduced cortical surface area in preterm children at seven years of age, which is closer to our result, albeit in a different age group. Zhang normalized their gyrification and surface area measures by convex hull area and total cortical surface area, respectively, while Engelhardt reported unnormalized group differences. Both normalization methods are fundamentally different from our analysis, in which we corrected for PMA at MRI and TTV. The differences in magnitude between our study and these previous studies can likely be explained by the injury status of the preterm cohort and the type of normalization performed.

Other works examining sulcal depth in very preterm subjects have reported just decreases^14^ or both increases and decreases at TEA^13^ and in childhood^15^, with decreases tending to dominate. Although there was no global difference between our two groups, there were more significant regional sulcal depth decreases (12) than increases (five) in the very preterm group, which is consistent with previous work.

Curvature was increased at TEA in our VPT group, which corroborates Makropoulos *et al*.’s findings^16^. Many different curvature formulations have been utilized, making this a challenging metric to compare to previous work. Curvature has been reported on both the outer cortical surface and on the inner cortical/outer white matter surface, as in the current work. It has been formulated as the mean of the principal curvatures as in the current work, Gaussian curvature, etc. (See Pienaar^45^ for an in-depth review). Given the larger number of regions with significant sulcal depth decrease in the very preterm group, immature sulci may still be driving increased curvature. Shallower sulci are missing sulcal wall area (low curvature), but have the same amount of sulcal trough area (high curvature), which results in higher average curvature in that local region^14^. The negative relationship between 1) sulcal depth and curvature and 2) gyrification index and curvature reinforces the idea that decreased folding may increase curvature in the very preterm group.

Our preterm only regression analyses confirmed BPD and ROP as clinical risk factors of abnormal cortical maturation in very preterm infants. A significant relationship persisted after controlling the final multivariable models for carefully selected clinical covariates. The negative relationship between BPD and surface area was widespread, involving all lobes of the brain (Table 4). Our lobar analysis also revealed that parietal lobe sulcal depth was the main driver of the negative relationship between this cortical metric and ROP (Table 5). Although BPD is a well-established, independent risk factor for NDI, no structural abnormalities except ventriculomegaly consistently explain the poor outcomes associated with BPD^27,46^. BPD and ROP both predicted NDI independently of structural injury in a large cohort of extremely preterm infants^21^, but our study is the first to link both to cortical maturational abnormalities. A few studies linked these antecedents to decreased white matter maturation, but none using the objective MRI measures of our study. Both BPD and ROP were associated in univariable analyses with lower overall maturation score (evaluated visually/qualitatively) on TEA MRI in preterm infants^47^, but only BPD remained significant in multivariable analyses. Both BPD and ROP share systemic inflammation and hyperoxia as common mechanisms that could disrupt brain development. It is challenging to determine whether the link between BPD/ROP and cortical dysmaturation is causal or a result of the most developmentally delayed infants requiring more intervention and support. However, in a rat pup model, hyperoxia caused BPD-like lung changes, ROP-like hypervascularization, and reduced brain surface area^48^, suggesting that BPD and ROP are interrelated and cause brain changes beyond those induced by prematurity. More work is needed to confirm similar causal effects in preterm infants.

Our study’s strengths include a population-based cohort, assessment of quantitative cortical maturation measurements, and many covariates collected and temporally examined. However, several limitations should be acknowledged. Despite the large number of possible covariates collected, residual confounding could have introduced bias. Nevertheless, the association we identified with BPD and cortical surface area was especially strong. Furthermore, because we assessed numerous metrics, some false discovery is inevitable, even after applying FDR correction. Given that we excluded severely brain injured subjects, including those with ventriculomegaly, the brain changes reported here may be less severe than in other brain injured preterm cohorts. Finally, any automated segmentation is inherently imperfect. The dHCP pipeline represents a substantial improvement over labor-intensive manual segmentation, however its creators acknowledge a 2% rate of significant error^32^.

We identified widespread cortical maturation abnormalities at TEA in very preterm infants compared to full-term infants. These cortical abnormalities, specifically surface area and sulcal depth, appear to be exacerbated by clinical risk factors, BPD and ROP, respectively. Larger studies are needed to validate the relationship of these antecedents with cortical dysmaturation. Further work should examine the predictive value of cortical dysmaturation for NDI in VPT infants. This will allow clinicians to assess risk of impairment early and provide targeted early interventions.

## Supplementary information

Supplementary Material

## Data Availability

The datasets generated during the current study are available from the corresponding author on reasonable request.
