# Author Correction: Retinopathy of Prematurity and Bronchopulmonary Dysplasia are Independent Antecedents of Cortical Maturational Abnormalities in Very Preterm Infants

**DOI:** 10.1038/s41598-021-86025-4

**Published:** 2021-03-23

**Authors:** Julia E. Kline, Venkata Sita Priyanka Illapani, Lili He, Mekibib Altaye, Nehal A. Parikh

**Affiliations:** 1 Perinatal Institute, Cincinnati Children’s Hospital Medical Center, Cincinnati, OH USA; 2 Department of Pediatrics, University of Cincinnati College of Medicine, Cincinnati, OH USA; 3 Center for Perinatal Research, The Research Institute at Nationwide Children’s Hospital, Columbus, OH USA; 4 Divison of Biostatistics, Cincinnati Children’s Hospital Medical Center, Cincinnati, OH USA

Correction to: *Scientific Reports* 10.1038/s41598-019-56298-x, published online 23 December 2019

The original version of this Article contained an error in the order of the Figures. Figures 1 and 2 were published as Figures 2 and 1. As a result, the Figure legends were incorrect.

The original Figures 1 and 2 and accompanying legends appear below.

**Figure 1:**
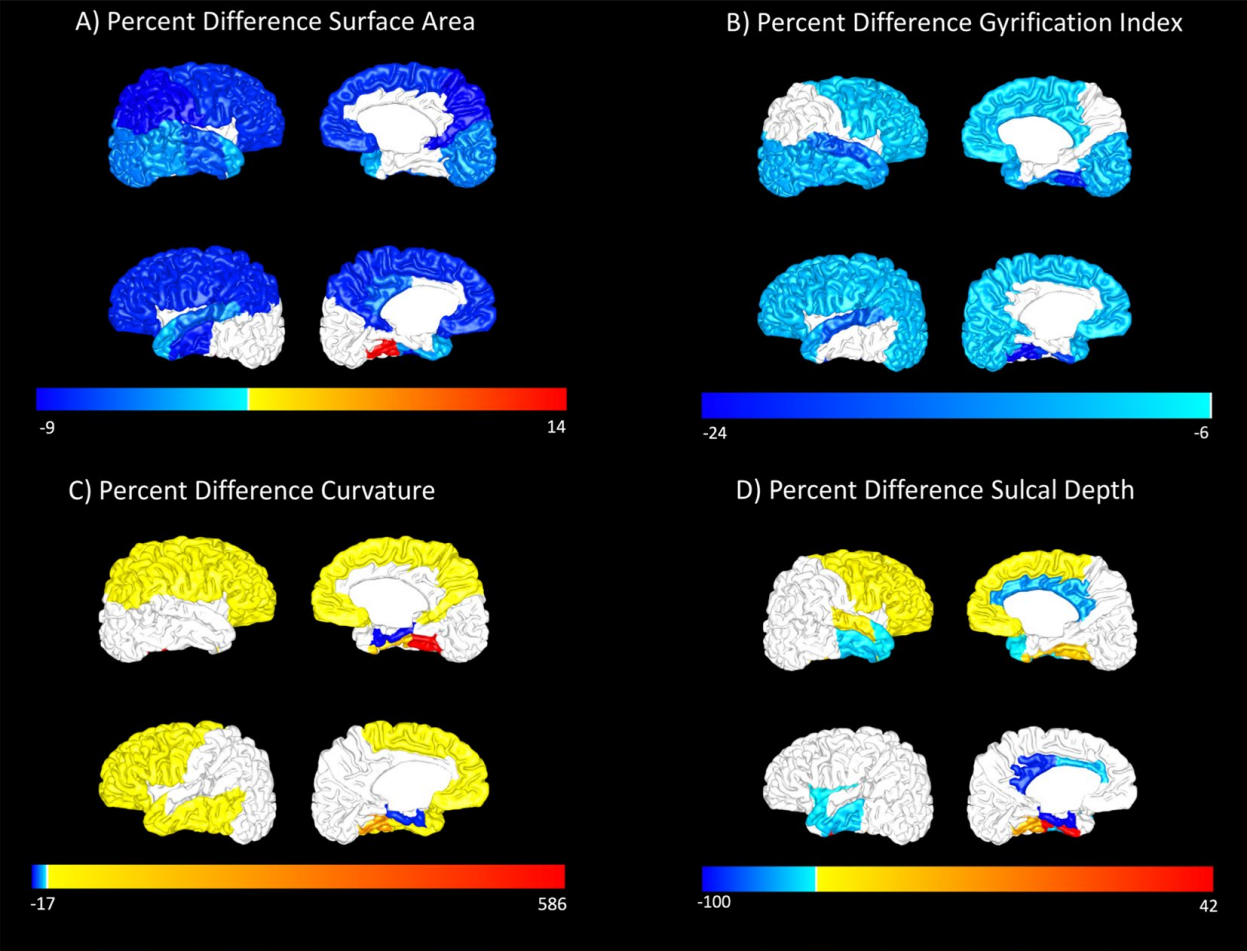
An example T2-weighted MRI scan from one VPT subject is shown in sagittal, coronal, and horizontal views (top row). A segmentation from the Developing Human Connectome Pipeline (dHCP) is overlaid on the original image (bottom row). The dHCP performs cortical and sub-cortical volume segmentation, cortical surface extraction, and cortical surface inflation and was specifically designed for neonatal T2-weighted MRI brain scans.

**Figure 2:**
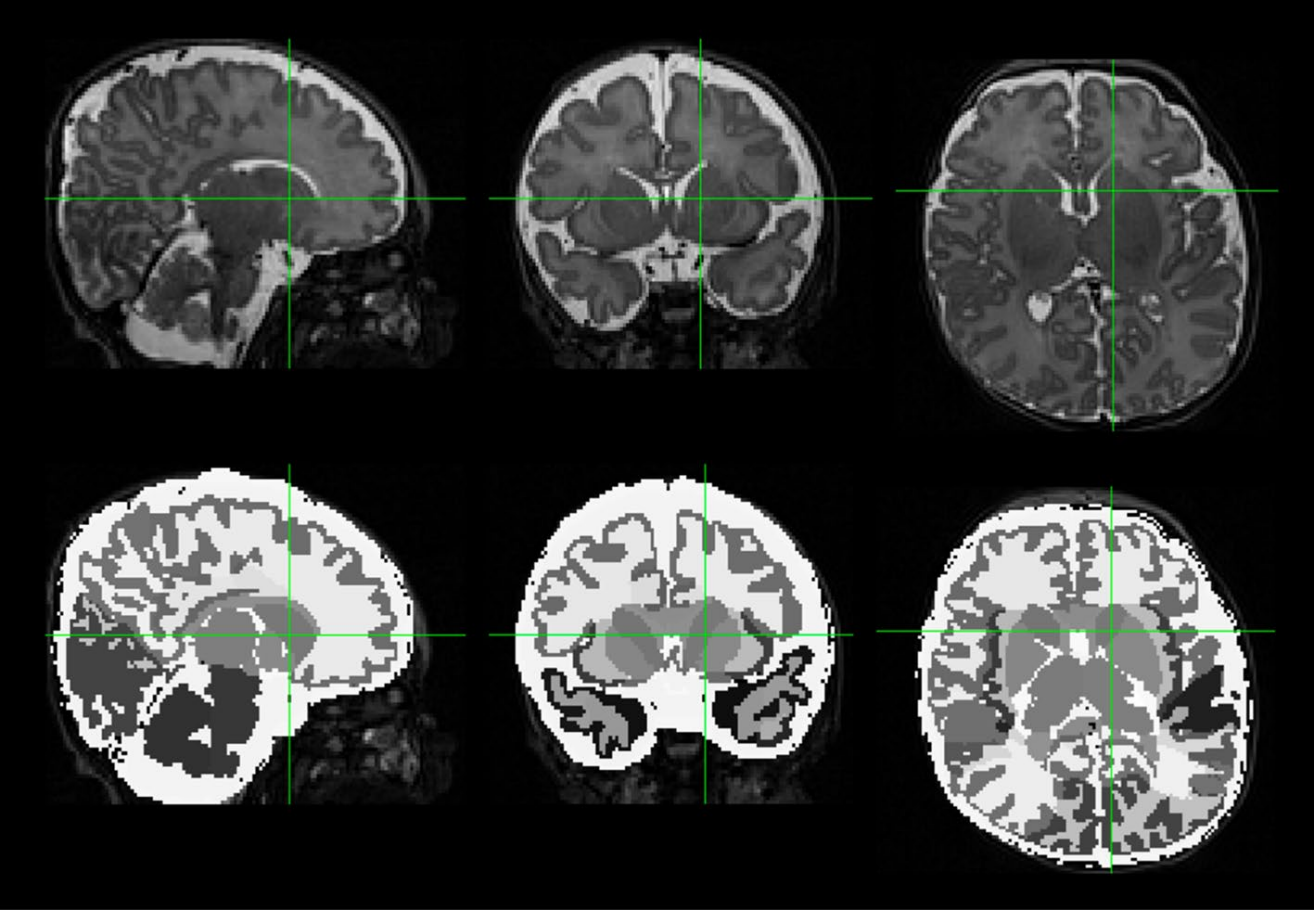
Mean Percent Difference in Cortical Metrics (Full-term to Very Preterm). Regional percent differences in adjusted group means for surface area (panel A), gyrification index (panel B), curvature of the white matter surface (panel C), and sulcal depth (panel D). For regions with significant differences between groups (after false discovery rate correction), percent difference values (VPT − FT)/FT * 100 are projected onto a representative subject brain from the very preterm group. These values have been corrected for postmenstrual age at MRI scan and total brain tissue volume. For each panel, Top row: right hemisphere; Bottom row: left hemisphere.

The original Article has been corrected.

