# Supplementary material for: Retinopathy of Prematurity and Bronchopulmonary Dysplasia are Independent Antecedents of Cortical Maturational Abnormalities in Very Preterm Infants
Source: Sci Rep. 2019 Dec 23;9:19679. doi: 10.1038/s41598-019-56298-x (PMC6928014; doi:10.1038/s41598-019-56298-x)
Supplement: Supplementary file 1 — Supplementary Material [file 41598_2019_56298_MOESM1_ESM.doc]

**Retinopathy of Prematurity and Bronchopulmonary Dysplasia are Independent Antecedents of Cortical Maturational Abnormalities in Very Preterm Infants**

Julia E. Kline, PhD^1^, Venkata Sita Priyanka Illapani, MS^1^, Lili He, PhD^1,2^, Mekibib Altaye, PhD^2,4^, Nehal A. Parikh, DO, MS*^1,2,3^

^1^Perinatal Institute, Cincinnati Children’s Hospital Medical Center, Cincinnati, OH

^2^Department of Pediatrics, University of Cincinnati College of Medicine, Cincinnati, OH

^3^Center for Perinatal Research, The Research Institute at Nationwide Children’s Hospital, Columbus, OH

^4^Divison of Biostatistics, Cincinnati Children’s Hospital Medical Center, Cincinnati, OH

*Corresponding author’s contact information:

Nehal A. Parikh, DO, MS

Professor of Pediatrics

Cincinnati Children’s Hospital

3333 Burnet Ave, MLC 7009

Cincinnati, OH 45229

(513) 636-7584 (Business)

(513) 803-0969 (Fax)

[Nehal.Parikh@cchmc.org](mailto:Nehal.Parikh@cchmc.org)

**Supplementary Table 1.** Whole-brain mean values and percent differences for five global cortical metrics in very preterm and full-term infants (corrected for PMA only).

| **Surface Metrics** | **Very Preterm Infants (N=94)** | **Full-term Infants (N=46)** | **Relative Percent Difference, Mean (95% CI)** | ***P*** |
| --- | --- | --- | --- | --- |
| **Gyrification Index** (unitless) | 2.49 (0.16) | 2.67 (0.17) | -6.74%  (-8.92%, -4.56%) | *<0.001* |
| **Surface Area**  (mm^2^) | 80724.9  (9569.37) | 87243.8  (10099.49) | -7.47%  (-11.45%, -3.40%) | *<0.001* |
| **Curvature (**1/mm**)** | 2.15 (0.21) | 2.01 (0.22) | 6.97%  (3.19%, 10.74%) | *0.002* |
| **Sulcal Depth** (unitless; mean convexity/concavity) | 24.48 (3.00) | 24.78 (3.17) | -1.21%  (-3.18%, 5.60%) | 0.62 |


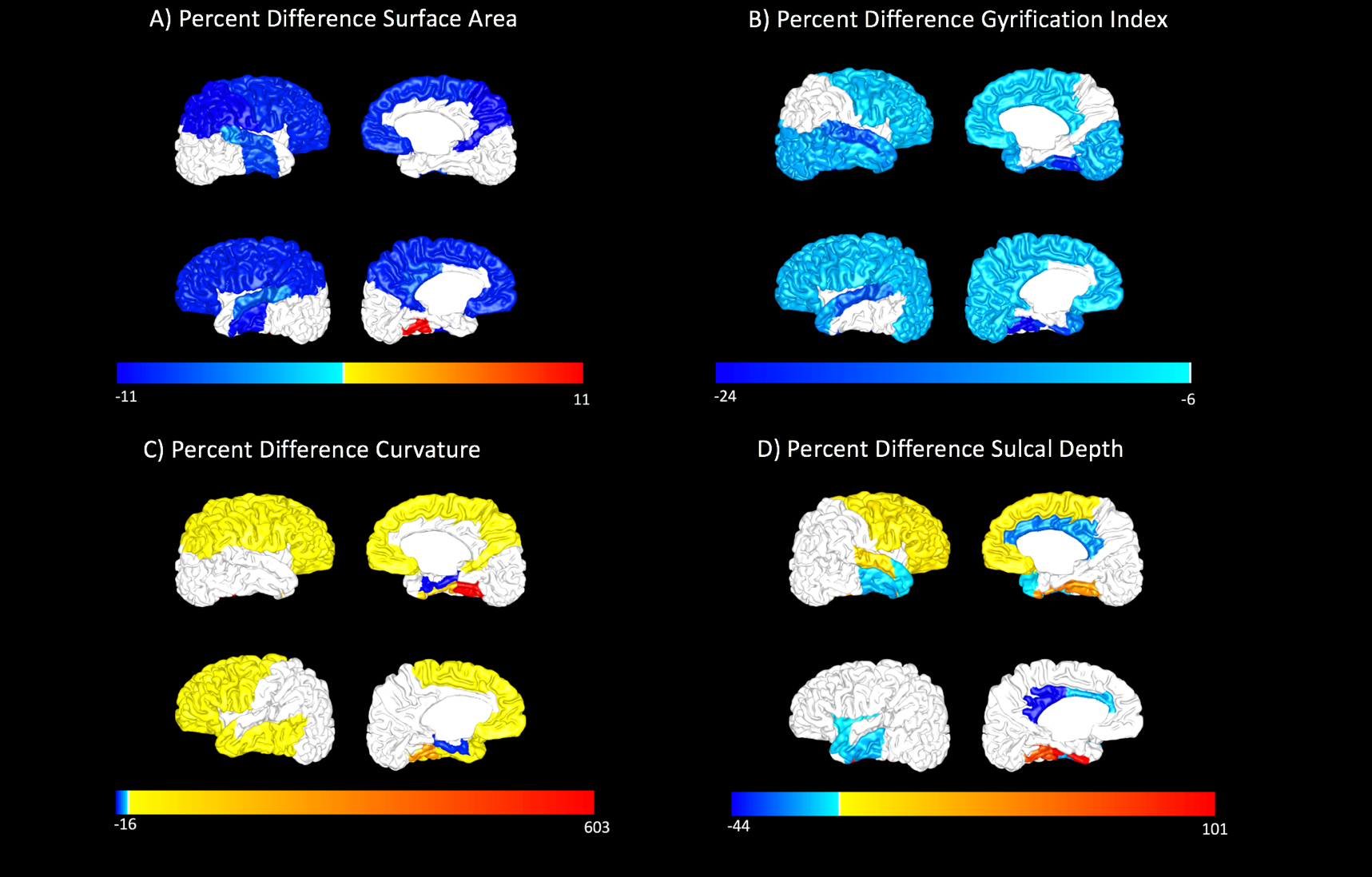


**Supplementary Figure 1: Mean Percent Difference in Cortical Metrics (Full-term to Very Preterm)**

Regional percent differences in adjusted group means for surface area (panel A), gyrification index (panel B), curvature of the white matter surface (panel C), and sulcal depth (panel D). For regions with significant differences between groups (after false discovery rate correction), percent difference values (VPT – FT)/FT*100 are projected onto a representative subject brain from the very preterm group. These values have been corrected for postmenstrual age at MRI scan. For each panel, Top row: right hemisphere; Bottom row: left hemisphere.
